# Desmoplakin mutation underlying autosomal dominant arrhythmogenic cardiomyopathy, palmoplantar keratoderma, and curly hair

**DOI:** 10.1016/j.jdcr.2023.03.018

**Published:** 2023-04-15

**Authors:** Colin Kincaid, Luke Horton, Brian Cheung, Ilhan Esse, Irmina Gradus-Pizlo, Natasha Atanaskova Mesinkovska

**Affiliations:** a Department of Dermatology, University of California Irvine, Irvine, California; b Department of Cardiology, University of California Irvine, Irvine, California; c Susan Samueli Integrative Health Institute, University of California, Irvine, California

**Keywords:** arrhythmogenic cardiomyopathy, cardiocutaneous syndrome, cardiomyopathy, Carvajal syndrome, desmoplakin, desmosome

## Introduction

Cardiocutaneous syndromes, characterized by a triad of cardiomyopathy, palmoplantar keratoderma (PPK), and woolly hair, have been linked to desmosomal gene mutations.^1^ Carvajal syndrome represents one such disorder that is classically caused by autosomal recessive (AR) mutations in the desmosomal component, desmoplakin (DSP). The associated cardiac symptoms can manifest early in life and, if unrecognized, may lead to sudden cardiac death.^2^ Here, we present a rare case of an autosomal dominant (AD) variant of Carvajal syndrome in a family in which subtle cutaneous features serve as warning signs of impending cardiac complications.

## Case report

**Fig 1:**
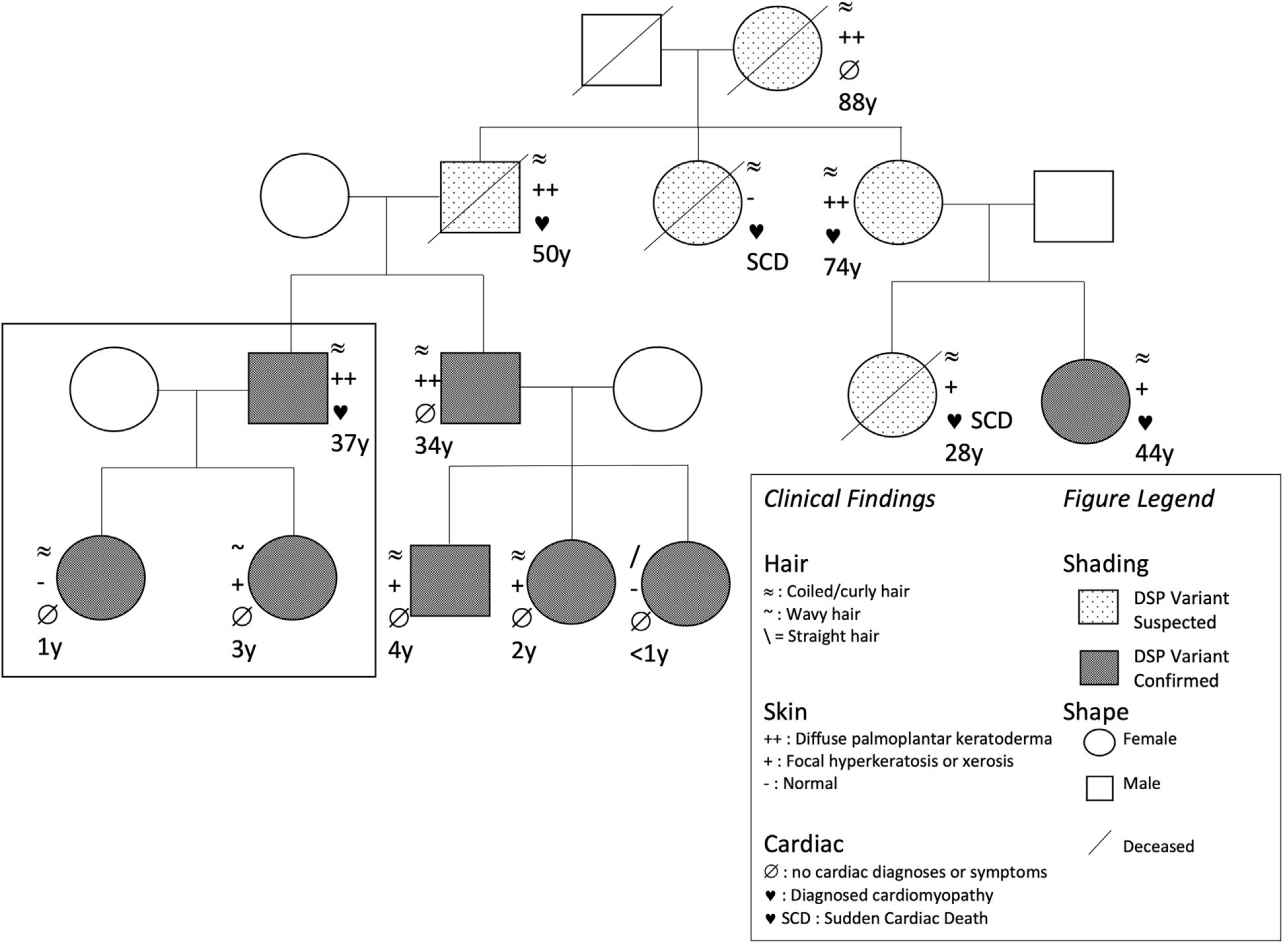
Pedigree of a family with an autosomal dominant variant of Carvajal syndrome. Desmoplakin variant confirmed using genetic testing. *DSP*, Desmoplakin; *SCD*, sudden cardiac death.

A 37-year-old man presented to his cardiologist with chest pain and palpitations. Seventeen years prior, he underwent placement of a preventative implantable cardioverter-defibrillator because of a family history significant for several generations of cardiomyopathy, ventricular arrhythmias, and/or sudden cardiac death (Fig 1). Despite his family history, the initial genetic testing results for familial cardiomyopathy in 2010 were nonrevealing. He remained asymptomatic since the placement of the implantable cardioverter-defibrillator, with a stable ejection fraction and ventricle size on annual transthoracic echocardiograms. The patient gave consent for their photographs and medical information to be published in print and online and with the understanding that this information may be publicly available.

**Fig 2:**
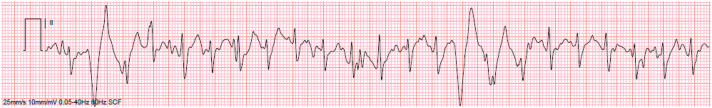
Cardiac stress test demonstrating nonsustained run of ventricular tachycardia.

Cardiac stress testing revealed nonsustained ventricular tachycardia (Fig 2) which prompted repeat genetic testing, this time revealing a heterozygous frameshift mutation (exon 19, c.2652_2653del, p. Gln884.HisfsX9) in the *DSP* gene. This mutation resulted in a premature stop codon in the N-terminal head domain of *DSP*, which, in the setting of arrhythmogenic cardiomyopathy, was suggestive of an AD variant of Carvajal syndrome. The same mutation was identified in 7 other family members (Fig 1), including his 1- and 3-year-old daughters.

**Fig 3:**
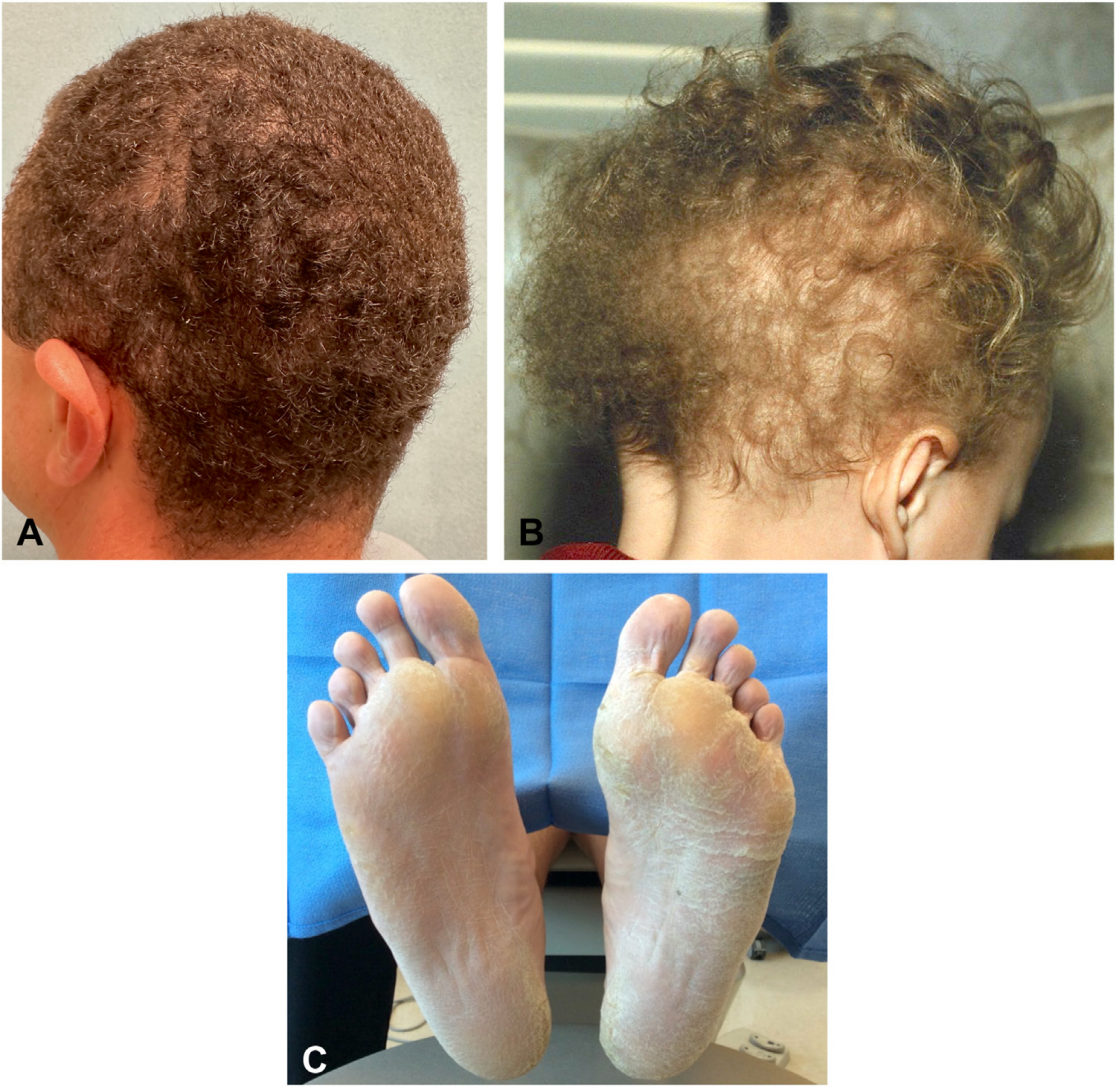
Tightly coiled/woolly hair of patients at ages (**A**) 37 years and (**B**) 16 months. **C,** Diffuse hyperkeratotic plantar plaques with bilateral fissuring.

**Fig 4:**
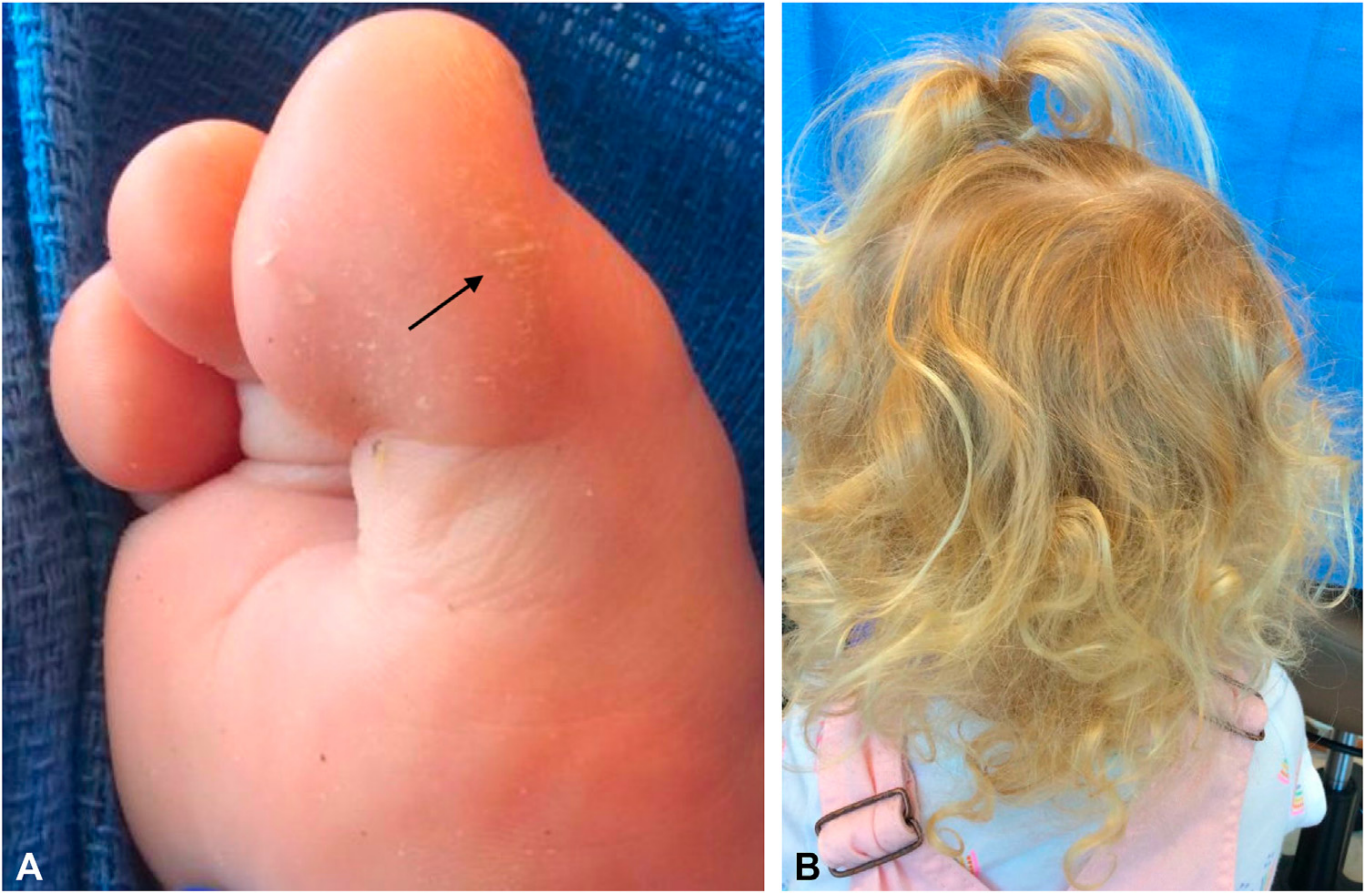
**A,** Small, focal hyperkeratotic plaque on the lateral aspect of the first toe of the daughter (*black arrow*). **B,** Fine, curly hair of the daughter.

As a part of the family workup for the cardiocutaneous syndrome, dermatology was involved in their clinical care. A clinical examination of his scalp revealed a closely shaved head of tightly coiled hair (Fig 3, *A*), which he endorsed had been present since birth (Fig 3, *B*), long before any cardiac symptoms. Additionally, he had diffuse hyperkeratotic plaques with fissures involving his palmar and plantar surfaces bilaterally (Fig 3, *C*). He confirmed that other family members with a history of cardiac disease had similar hair and skin findings. On examination, his 3-year-old daughter had focal hyperkeratotic plaques bilaterally involving the plantar surfaces of her feet (Fig 4, *A*). She had fine, wavy-to-curly, blonde hair rather than the woolly hair of her father’s (Fig 4, *B*). His daughter is currently being followed by pediatric cardiology and has no cardiac abnormalities to date.

## Discussion

Since first being described in 1996, Carvajal syndrome has primarily been reported as an AR cardiocutaneous disorder, although rare AD cases have more recently been reported.^1^^,^^3^, ^4^, ^5^ The pathogenesis involves mutations in the *DSP* gene which encodes the most abundant desmosomal protein, desmoplakin.^2^ Desmoplakin plays an important role in maintaining tissue integrity by adhering neighboring cells through interactions with intermediate filaments such as desmin and keratin.^3^ The abundant distribution of DSP in the heart and epidermis underlies the cardiocutaneous phenotype, classically manifesting as a triad of striate PPK, woolly hair, and cardiomyopathy. Variations of this phenotype, with additional clinical features such as dental abnormalities and leukonychia, support a degree of phenotypic heterogeneity across DSP variants.^6^^,^^7^ Recent reports of AD variants have given rise to inconsistent nomenclature, with cases reported as either an AD variant of Carvajal syndrome^6^^,^^7^ or a distinct entity with arrhythmogenic cardiomyopathy and Carvajal-like features.^1^^,^^3^^,^^5^

The characteristic cutaneous features are typically the earliest clinical signs, with woolly hair usually present at birth and PPK appearing later in adolescence.^2^ The cardiac phenotype and age at presentation appear to depend on the variant. The most common cardiac feature of the recessive variant is dilated cardiomyopathy, which often presents as heart failure or ventricular arrhythmia during early childhood.^2^ The dominant variant is more associated with arrhythmogenic cardiomyopathy that presents during adulthood,^1^ although cardiac symptoms have been reported at as young as 10 years of age.^7^ In our patient’s family, those with cardiac involvement first experienced symptoms between 28 and 37 years of age. The associated cardiac features include inverted T-waves, nonsustained ventricular tachycardia on stress testing, ventricular dilation on echocardiography, and fibrofatty infiltration of the epicardium on cardiac magnetic resonance imaging.^1^^,^^2^ If unrecognized, cardiac symptoms may progress to sudden cardiac death due to lethal arrhythmia, highlighting the need for early identification and intervention.

Although the patient’s 3-year-old daughter carries the same mutation, her cutaneous findings do not perfectly align with the abovementioned classical triad, reflecting phenotypic differences among the variants of Carvajal syndrome.^1^^,^^5^ A recent study of 6 families with AD variant *DSP* mutations demonstrated a less pronounced phenotype compared with the classical AR variants.^1^ The affected individuals had curly or wavy hair (rather than woolly) and hyperkeratosis ranging from focal to diffuse plantar plaques. Interestingly, nearly all patients with AD mutations had curly hair, whereas family members without these mutations had noncurly hair, suggesting that curls may help identify affected individuals.^1^ Another study of 14 heterozygous probands and their family members reported a highly penetrant phenotype of curly hair and PPK with variable severity, whereas only 60% had a cardiac phenotype.^5^ However, the severity of cutaneous disease was not predictive of the presence nor severity of cardiac disease.^5^ This suggests that although the daughter did not have striking cutaneous findings, she still remains at risk of developing cardiomyopathy.

Given that cutaneous findings precede cardiac symptoms, it is important to recognize the constellation of curly/woolly hair and PPK as a possible connection to cardiac abnormalities. Because these findings may be subtle, cardiocutaneous syndromes should not be ruled out when classical severe features are not appreciated, especially in the presence of a family history of cardiomyopathy or sudden cardiac death. Genetic testing and referral to cardiology in any extended family members demonstrating similar cutaneous phenotypes, regardless of severity, should be considered.

## Conflicts of interest

None disclosed.
